# Data on iron and turbidity in a drip irrigation system in New Jersey, USA

**DOI:** 10.1016/j.dib.2019.01.038

**Published:** 2019-01-19

**Authors:** Steven Yergeau, Amy Raudenbush

**Affiliations:** a Rutgers Cooperative Extension of Ocean & Atlantic Counties, 1623 Whitesville Road, Toms River NJ 08755, United States; b The Ohio State University, United States

## Abstract

The dataset contains irrigation water quality parameters including iron concentrations (ferrous, ferric, and total) and turbidity measurements from a blueberry farm in southern New Jersey. Data was collected from May through June for 14 weeks in 2015 using a SMART3 Colorimeter™. Water samples were taken from the pump at the irrigation well and compared to samples taken from driplines at individual fields. All samples were analyzed in the field following the equipment׳s manual, except for ferric iron, which was calculated from the ferrous iron and total iron concentrations. Statistical analyses were performed on the dataset to understand the data. This data is being made available to increase the understanding of clogging in drip irrigation systems and to assist in the management of drip irrigation systems.

**Specifications table**

**Table:** 

Subject area	*Environmental Chemistry, Earth Science*
More specific subject area	*Agricultural Water Quality, Irrigation Management*
Type of data	*The data is presented in three (3) tables and four (4) figs.*
How data was acquired	*SMART3 Colorimeter™ with reagent system for 1,10-Phenanthroline Method for measuring iron and the Absorption Method for turbidity were used in the field to collect data.*
Data format	*Raw and analyzed*
Experimental factors	*Prior to sample collection, water from both the pump and field lines were allowed to run freely for one to two minutes. Each collection bottle was rinsed three times with sample water before samples were taken for analyses.*
Experimental features	*Iron (ferrous iron and total iron) and turbidity were measured at the pump and at the end of lines in a drip irrigation system at a blueberry farm. Ferric iron was calculated from the ferrous iron and total iron concentrations obtained.*
Data source location	*Blueberry farm in Egg Harbor City, Atlantic County, New Jersey, USA. The farm is located at coordinates latitude 39.565132, longitude -74.679929.*
Data accessibility	*Data is contained within this article.*
Related research article	*Chauhdary, J.N., A. Bakhsh, N. Ahmad, and K. Mehmood. (2015). Optimizing chlorine use for improving performance of drip irrigation system under biologically contaminated water source. Pakistan Journal of Agricultural Sciences, 52(3), 829–835.*

**Value of the data**

• The dataset can be used to monitor iron concentrations and turbidity in farm irrigation water.
• Knowledge of iron content and turbidity can assist decisions on the treatment of water for clogging in drip irrigation systems.
• Knowledge of clogging will help with proper management for drip irrigation systems and help pinpoint the causes and target solutions.
• The data presented can help growers save costs on managing drip irrigation systems.

## 1 Data

**Fig. 1:**
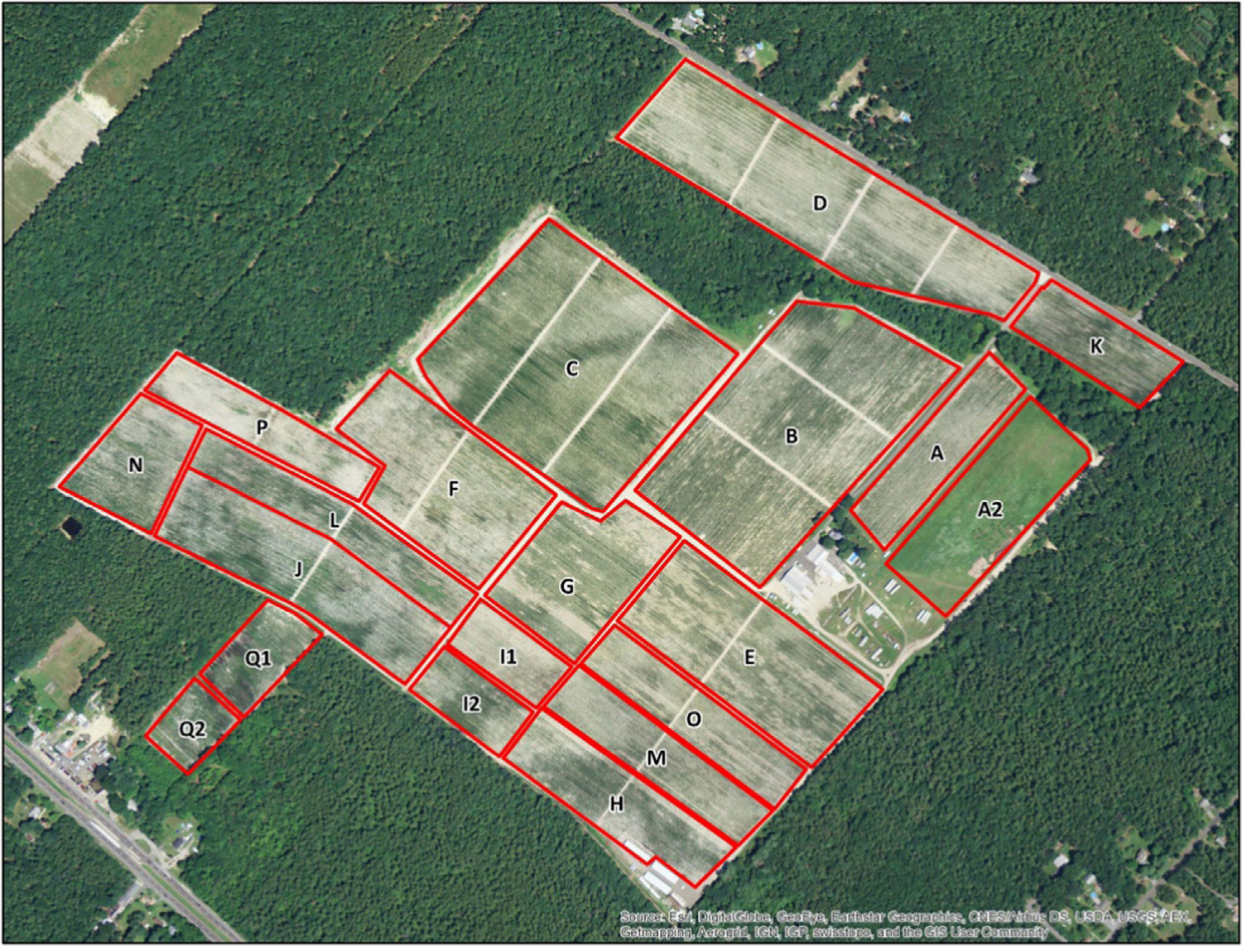
Map of blueberry farm monitored as part of this project showing the active, growing fields. (Each field is outlined in red and designated with a letter to help schedule irrigation).

**Fig. 2:**
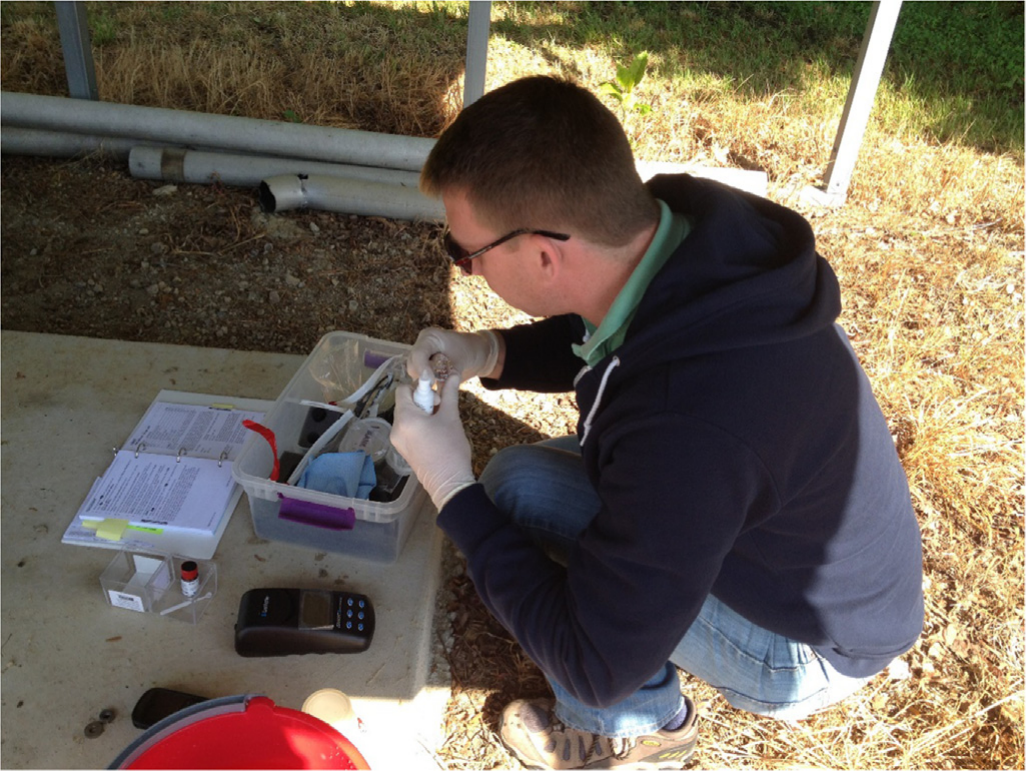
Use of the LaMotte SMART3 Colorimeter™ during sample analysis.

**Fig. 3:**
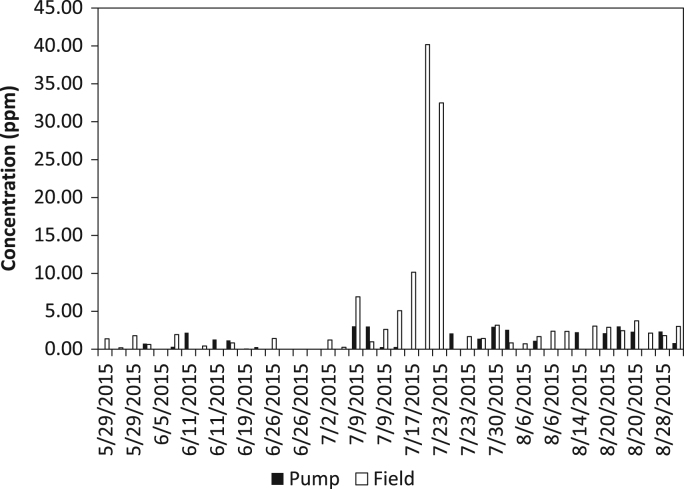
Graph of turbidity levels measured at the pump and in the field.

**Fig. 4:**
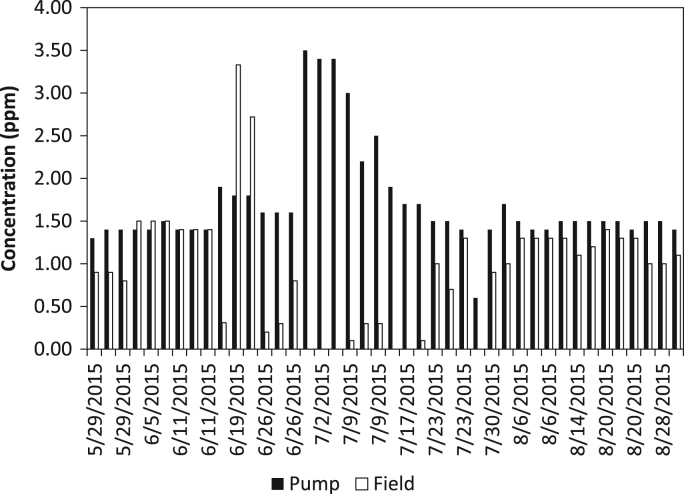
Graph of ferrous iron levels measured at the pump and in the field.

**Fig. 5:**
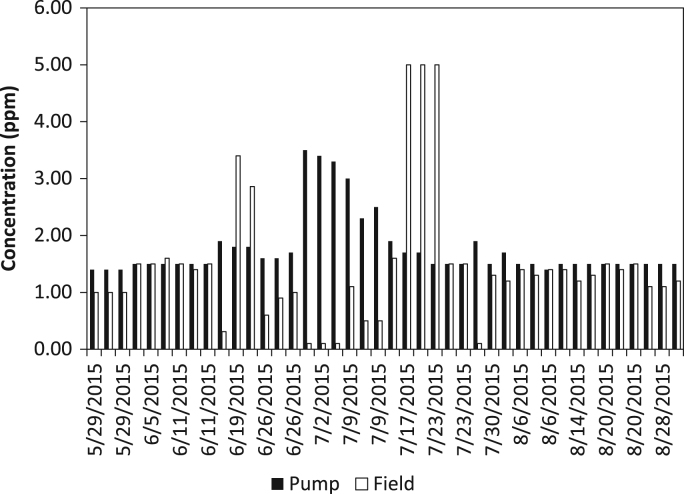
Graph of total iron levels measured at the pump and in the field.

**Fig. 6:**
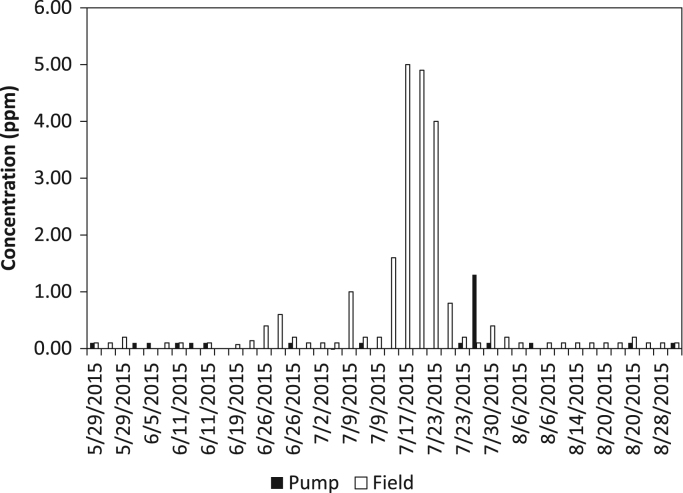
Graph of ferric iron levels measured at the pump and in the field.

The dataset contains water quality of irrigation water from a Highbush blueberry (*Vaccinium corymbosum*) agricultural farm in Egg Harbor City, Atlantic County, New Jersey, USA (Fig. 1). The water quality parameters collected include iron concentration (ferrous, ferric, and total iron) and turbidity. All water samples were analyzed in the field using a SMART3 Colorimeter™ (Fig. 2) and the appropriate reagents following the procedures outlined in the operator׳s manual [1]. The dataset is shown in Tables 1 and 2, and in Fig. 3, Fig. 4, Fig. 5, Fig. 6. Understanding iron concentrations will aid with proper irrigation and water management, which in turn will assist with optimizing water usage to improve crop productivity [2]. The descriptive statistics to further understand the data are shown in Table 3.

**Table 1:** Turbidity, ferrous iron, total iron, and ferric iron concentrations from the pump samples.

**Location ID**	**Date**	**Turbidity (FAU)**	**Ferrous Iron (ppm)**	**Total Iron (ppm)**	**Ferric Iron (ppm)**^a^
Pump	5/22/2015	0.00	1.80	1.90	0.10
Pump	5/22/2015	0.00	1.90	1.90	0.00
Pump	5/22/2015	0.00	1.90	2.10	0.20
Pump	5/29/2015	0.00	1.30	1.40	0.10
Pump	5/29/2015	0.00	1.40	1.40	0.00
Pump	5/29/2015	0.00	1.40	1.40	0.00
Pump	6/5/2015	0.74	1.40	1.50	0.10
Pump	6/5/2015	0.00	1.40	1.50	0.10
Pump	6/5/2015	0.32	1.50	1.50	0.00
Pump	6/11/2015	2.18	1.40	1.50	0.10
Pump	6/11/2015	0.00	1.40	1.50	0.10
Pump	6/11/2015	1.27	1.40	1.50	0.10
Pump	6/19/2015	1.17	1.90	1.90	0.00
Pump	6/19/2015	0.00	1.80	1.80	0.00
Pump	6/19/2015	0.27	1.80	1.80	0.00
Pump	6/26/2015	0.00	1.60	1.60	0.00
Pump	6/26/2015	0.00	1.60	1.60	0.00
Pump	6/26/2015	0.00	1.60	1.70	0.10
Pump	7/2/2015	0.00	3.50	3.50	0.00
Pump	7/2/2015	0.00	3.40	3.40	0.00
Pump	7/2/2015	0.00	3.40	3.30	−0.10
Pump	7/9/2015	3.02	3.00	3.00	0.00
Pump	7/9/2015	3.00	2.20	2.30	0.10
Pump	7/9/2015	0.28	2.50	2.50	0.00
Pump	7/17/2015	0.29	1.90	1.90	0.00
Pump	7/17/2015	0.00	1.70	1.70	0.00
Pump	7/17/2015	0.00	1.70	1.70	0.00
Pump	7/23/2015	0.00	1.50	1.50	0.00
Pump	7/23/2015	2.08	1.50	1.50	0.00
Pump	7/23/2015	0.00	1.40	1.50	0.10
Pump	7/30/2015	1.39	0.60	1.90	1.30
Pump	7/30/2015	2.96	1.40	1.50	0.10
Pump	7/30/2015	2.55	1.70	1.70	0.00
Pump	8/6/2015	0.00	1.50	1.50	0.00
Pump	8/6/2015	1.10	1.40	1.50	0.10
Pump	8/6/2015	0.00	1.40	1.40	0.00
Pump	8/14/2015	0.00	1.50	1.50	0.00
Pump	8/14/2015	2.24	1.50	1.50	0.00
Pump	8/14/2015	0.00	1.50	1.50	0.00
Pump	8/20/2015	2.11	1.50	1.50	0.00
Pump	8/20/2015	3.01	1.50	1.50	0.00
Pump	8/20/2015	2.31	1.40	1.50	0.10
Pump	8/28/2015	0.00	1.50	1.50	0.00
Pump	8/28/2015	2.34	1.50	1.50	0.00
Pump	8/28/2015	0.80	1.40	1.50	0.10

a Ferric iron was calculated as total iron concentration minus ferrous iron concentration.

**Table 2:** Turbidity, ferrous iron, total iron, and ferric iron concentrations from the field samples. Each field letter designation corresponds to the fields at the farm (see Fig. 1).

**Location ID**	**Date**	**Turbidity (FAU)**	**Ferrous Iron (ppm)**	**Total Iron (ppm)**	**Ferric Iron (ppm)**^a^
Field M	5/29/2015	1.37	0.90	1.00	0.10
Field M	5/29/2015	0.19	0.90	1.00	0.10
Field M	5/29/2015	1.78	0.80	1.00	0.20
Field D	6/5/2015	0.62	1.50	1.50	0.00
Field D	6/5/2015	0.00	1.50	1.50	0.00
Field D	6/5/2015	1.92	1.50	1.60	0.10
Field B	6/11/2015	0.00	1.40	1.50	0.10
Field B	6/11/2015	0.42	1.40	1.40	0.00
Field B	6/11/2015	0.00	1.40	1.50	0.10
Field B	6/19/2015	0.83	0.31	0.31	0.00
Field B	6/19/2015	0.03	3.33	3.40	0.07
Field B	6/19/2015	0.00	2.72	2.86	0.14
Field D	6/26/2015	1.41	0.20	0.60	0.40
Field D	6/26/2015	0.00	0.30	0.90	0.60
Field D	6/26/2015	0.00	0.80	1.00	0.20
Field O	7/2/2015	0.00	0.00	0.10	0.10
Field O	7/2/2015	1.21	0.00	0.10	0.10
Field O	7/2/2015	0.25	0.00	0.10	0.10
Field D	7/9/2015	6.91	0.10	1.10	1.00
Field D	7/9/2015	0.98	0.30	0.50	0.20
Field D	7/9/2015	2.62	0.30	0.50	0.20
Field D	7/17/2015	5.08	0.00	1.60	1.60
Field D	7/17/2015	10.16	0.00	5.00	5.00
Field D	7/17/2015	40.18	0.10	5.00	4.90
Field B	7/23/2015	32.48	1.00	5.00	4.00
Field B	7/23/2015	0.00	0.70	1.50	0.80
Field B	7/23/2015	1.67	1.30	1.50	0.20
Field L	7/30/2015	1.41	0.00	0.10	0.10
Field L	7/30/2015	3.17	0.90	1.30	0.40
Field L	7/30/2015	0.83	1.00	1.20	0.20
Field E	8/6/2015	0.71	1.30	1.40	0.10
Field E	8/6/2015	1.67	1.30	1.30	0.00
Field E	8/6/2015	2.37	1.30	1.40	0.10
Field N	8/14/2015	2.35	1.30	1.40	0.10
Field N	8/14/2015	0.00	1.10	1.20	0.10
Field N	8/14/2015	3.04	1.20	1.30	0.10
Field A2	8/20/2015	2.89	1.40	1.50	0.10
Field A2	8/20/2015	2.45	1.30	1.40	0.10
Field A2	8/20/2015	3.74	1.30	1.50	0.20
Field J	8/28/2015	2.13	1.00	1.10	0.10
Field J	8/28/2015	1.80	1.00	1.10	0.10
Field J	8/28/2015	3.01	1.10	1.20	0.10

a Ferric iron was calculated as total iron concentration minus ferrous iron concentration.

**Table 3:** Summary of the descriptive statistics of the data.

**Location**	**Parameter**	**Mean ± SE**	**Median**	**Mode**	**Std Dev**	**Sample Var**	**Kurtosis**	**Skewness**	**Range**	**Min**	**Max**
Pump	Ferric Iron (ppm)	0.06 ± 0.03	0.00	0.00	0.20	0.04	36.55	5.86	1.40	−0.10	1.30
Pump	Ferrous Iron (ppm)	1.71 ± 0.09	1.50	1.40	0.60	0.36	3.65	1.93	2.90	0.60	3.50
Pump	Total Iron (ppm)	1.77 ± 0.09	1.50	1.50	0.55	0.31	4.10	2.25	2.10	1.40	3.50
Pump	Turbidity (FAU)	0.84 ± 0.17	0.14	0.00	1.10	1.21	-0.77	0.92	3.02	0.00	3.02
Field	Ferric Iron (ppm)	0.53 ± 0.18	0.10	0.10	1.20	1.43	9.36	3.20	5.00	0.00	5.00
Field	Ferrous Iron (ppm)	0.93 ± 0.11	1.00	1.30	0.71	0.50	2.33	0.95	3.33	0.00	3.33
Field	Total Iron (ppm)	1.46 ± 0.18	1.30	1.50	1.17	1.38	4.28	2.02	4.90	0.10	5.00
Field	Turbidity (FAU)	3.37 ± 1.20	1.41	0.00	7.77	60.34	16.60	4.06	40.18	0.00	40.18

## 2 Experimental design, materials, and methods

### 2.1 Sampling site

A blueberry farm in Egg Harbor City (Atlantic County, NJ) was monitored in 2015. The farm has 20 individual blueberry fields that cover approximately 250 acres, containing three varieties of Highbush blueberries (*Vaccinium corymbosum*): Duke, Bluecrop, and Elliot (Fig. 1). The irrigation system is a drip line fed by a single well where a pump delivered water to fields where fertigation occurred, as needed, to minimize the loss of fertilizers, pesticides, and other chemicals as the water travels through the system. The components of the drip irrigation system at this farm are controlled by a computer system to allow greater control over the watering schedule. Fields were irrigated on a rotating basis, determined by previous rainfall, soil moisture, and plant need.

### 2.2 Sample collection

Samples were collected over 14 weeks from May through August 2015, during the active growing and harvesting season for blueberries in New Jersey. Water samples were collected at both the irrigation well pump and at the end of the drip line in several fields. Sample bottles for collecting water from the pump were kept separate from bottles used to collect field water to avoid cross contamination. Three samples from the irrigation pump and three samples from the field were collected for analysis of iron content and turbidity. Pump samples were collected in bottles with water from a spigot attached to the side of the pump. Field samples were collected by opening the end of one of the drip lines at the end of a row of blueberry bushes and letting the water flow into a sample bottle. All bottles were rinsed three times with sample water (either from the pump or field) before collection to prevent cross contamination from previous sample collection. The drip lines were sampled randomly on the day of sampling. Fields sampled were determined based upon which fields were undergoing active irrigation on the day of sampling.

### 2.3 Sample analysis

All samples taken from the irrigation pump and field drip lines were analyzed for iron (ferrous [soluble] and total iron) and turbidity using a LaMotte SMART3 Colorimeter™ (Chestertown, Maryland) with the appropriate iron reagents. The SMART3 Colorimeter™ was chosen for its accuracy, durability, and its ability to provide cost-effective, fast, and reliable results as a portable piece of field equipment. The analytical methods for iron (1,10-Phenanthroline Method, Code 3668-SC) and turbidity (Absorption Method) were followed as outlined in the LaMotte SMART3 Colorimeter™ Operator׳s Manual [1]. Analyses measured concentrations for ferrous iron and total iron as ppm, and turbidity levels as Formazon Attenuation Units (FAUs) (Table 1 and Table 2). Ferric (oxidized) iron concentration was calculated as the remainder when ferrous iron data were subtracted from total iron (Tables 1 and 2).

### 2.4 Descriptive statistics

Descriptive statistics for iron content and turbidity in the pump water and the field irrigation water were calculated using the statistical software package in Microsoft Excel™ (Table 3).
